# Who can safely discontinue lifelong follow‐up among patients with sporadic pheochromocytoma and paraganglioma?

**DOI:** 10.1111/joim.70080

**Published:** 2026-03-04

**Authors:** Min Jeong Park, Seung Shin Park, Won Woong Kim, Su‐Jin Kim, Yu‐Mi Lee, Kyu Eun Lee, Tae‐Yon Sung, Jae‐Kyung Won, Dong Eun Song, Jung‐Min Koh, Sara Talvacchio, Tamara Prodanov, Hussam Alkaissi, Karel Pacak, Seung Hun Lee, Jung Hee Kim

**Affiliations:** ^1^ Department of Internal Medicine Korea University Medical Center Guro Hospital Seoul Republic of Korea; ^2^ Department of Internal Medicine Seoul National University Hospital Seoul Republic of Korea; ^3^ Department of Internal Medicine Seoul National University College of Medicine Seoul Republic of Korea; ^4^ Department of Surgery Asan Medical Center, University of Ulsan College of Medicine Seoul Republic of Korea; ^5^ Department of Surgery Seoul National University College of Medicine Seoul Republic of Korea; ^6^ Department of Pathology Seoul National University College of Medicine Seoul Republic of Korea; ^7^ Department of Pathology Asan Medical Center, University of Ulsan College of Medicine Seoul Republic of Korea; ^8^ Division of Endocrinology and Metabolism, Department of Internal Medicine Asan Medical Center, University of Ulsan College of Medicine Seoul Republic of Korea; ^9^ AKESO Center for Adrenal Endocrine Tumors Prague Czech Republic; ^10^ Eunice Kennedy Shriver National Institute of Child Health and Human Development Bethesda Maryland USA; ^11^ Faculty of Medicine Palacky University Olomouc Czech Republic; ^12^ 5th Department of Internal Medicine, Faculty of Medicine Comenius University Bratislava Slovakia; ^13^ National Institute of Diabetes and Digestive and Kidney Diseases Bethesda Maryland USA

**Keywords:** criteria, follow‐up, metastasis, paraganglioma, pheochromocytoma, recurrence

## Abstract

**Background:**

Current guidelines recommend at least 10 years of follow‐up for all pheochromocytoma and paraganglioma (PPGL) patients and lifelong monitoring for high‐risk individuals. Nonetheless, data identifying patients who may not require routine lifelong follow‐up are scarce.

**Methods:**

Among 999 patients with PPGL, 703 who were non‐metastatic, non‐hereditary, and had undergone complete resection were included. Variables that significantly differed between the recurrence (*n* = 50) and non‐recurrence groups over 10 years (*n* = 83) were identified, and cutoff values were determined using receiver‐operating characteristic curve analysis. These very low‐risk criteria were validated in an internal cohort and an external dataset from the National Institutes of Health.

**Results:**

The non‐recurrence group was older and had smaller pheochromocytomas (PCCs) than the recurrence group, with cutoffs of 37 years and 5.7 cm, respectively. The non‐recurrence group had a higher percentage of patients with pheochromocytoma of the adrenal gland scaled score (PASS) <4 or grading system for adrenal pheochromocytoma and paraganglioma (GAPP) score <3 (*p *= 0.027). Age >40 years (hazard ratio [HR] [95% confidence intervals] of 0.36 [0.17–0.76]), PCC size <6 cm (HR = 0.43 [0.19–0.98]), and PASS <4 or GAPP score <3 (HR = 0.37 [0.16–0.89]) were associated with lower recurrence risk. None of the patients meeting all these criteria in the internal (*n* = 114) and external (*n* = 13) validation sets experienced recurrence.

**Conclusion:**

This study suggests that routine lifelong follow‐up may be unnecessary for patients with sporadic PCC aged >40 years, size <6 cm, and PASS <4 or GAPP score <3.

## Graphical abstract

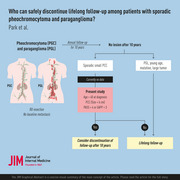

**Abbreviations**
AUROC: area under the receiver operating characteristic curve
CI: confidence interval
CT: computed tomography
DOTATOC‐PET: ^68^Ga‐labeled DOTA0‐Tyr3 octreotide positron emission tomography
FDG‐PET: ^18^F‐labeled fluorodeoxyglucose positron emission tomography
GAPP: grading system for adrenal pheochromocytoma and paraganglioma
HNPGL: head and neck paraganglioma
HR: hazard ratio
IQR: interquartile range
KADS: Korean Adrenal Disorder Study
MIBG: metaiodobenzylguanidine
MRI: magnetic resonance imaging
NIH: National Institutes of Health
PASS: pheochromocytoma of the adrenal gland scaled score
PCC: pheochromocytoma
PET: positron emission tomography
PGL: paraganglioma
PPGL: pheochromocytoma and paraganglioma
PV: pathogenic variant
ROC: receiver operating characteristic
SPECT: single‐photon emission computed tomography
WHO: World Health Organization

## Introduction

Pheochromocytomas and paragangliomas (PPGLs) are rare neuroendocrine tumors that originate from the chromaffin cells of the adrenal medulla (pheochromocytoma, PCC) or extra‐adrenal paraganglionic tissue (paraganglioma, PGL) [1]. PPGLs typically present with symptoms such as paroxysmal hypertension, arrhythmias, heart failure, or even mortality, often resulting from catecholamine overproduction, which underscores the urgency for effective treatment [2, 3]. The recurrence rates of PPGLs vary significantly, ranging from 3% to 20%, depending on the study population and duration of follow‐up [4, 5, 6, 7, 8]. Recurrence is associated with elevated morbidity and mortality [9]. Genetic mutations are known to double the risk of recurrence, with approximately 40% of PPGLs demonstrating a genetic predisposition [10, 11, 12]. Additionally, large tumor size, younger age at diagnosis, and PGL are associated with a higher recurrence risk [4, 13, 14].

Recurrence may occur decades after surgical resection [15]. Although the incidence of recurrence does not significantly decline after 5 years of follow‐up, the estimates beyond 10 years remain uncertain [14]. Consequently, recent guidelines recommend a minimum of 10 years of follow‐up for all patients after surgical resection [13, 14]. Patients at high risk of recurrence due to factors such as genetic predisposition, larger tumors, younger age at diagnosis, and/or a diagnosis of PGL are advised to undergo lifelong annual follow‐up [13, 14]. However, the necessity of lifelong surveillance for all patients with PPGLs has been increasingly challenged, as this practice imposes considerable burdens on patients and healthcare systems and may lead to unnecessary anxiety, medical interventions, and economic costs.

Although long‐term follow‐up is clearly warranted for patients with PPGLs carrying genetic predispositions, the necessity in patients with sporadic tumors remains unclear. A systematic review estimated a 5‐year recurrence probability of 4.7%–7.0% [4], whereas a meta‐analysis reported a prevalence of <3% among patients with sporadic PPGL [6]. These findings suggest that some subgroups of patients with sporadic PPGL may exhibit a low risk of recurrence or metastasis. This raises the possibility that lifelong follow‐up may not be necessary for all patients with sporadic PPGLs. Guidelines for the long‐term follow‐up of patients treated for a PPGL should consider not only the recurrence risk but also the psychological and financial burden of follow‐ups based on the recurrence incidence and the assessment of potential prognostic factors [13, 14]. However, current guidelines indicate that there are insufficient data to recommend follow‐up beyond 10 years for patients with sporadic small (<5 cm) PCC [13, 14], emphasizing the necessity for further data on recurrence patterns and pertinent prognostic factors.

In this study, we aimed to (1) examine the recurrence distribution of sporadic PPGL in a large, multicenter cohort and (2) identify a low‐risk subgroup of patients with no recurrence during a follow‐up period of 10 years or more, suggesting potential candidates for discontinuing lifelong follow‐up, and validate these findings in an independent PPGL cohort.

## Methods

### Study subjects and design

**Fig. 1:**
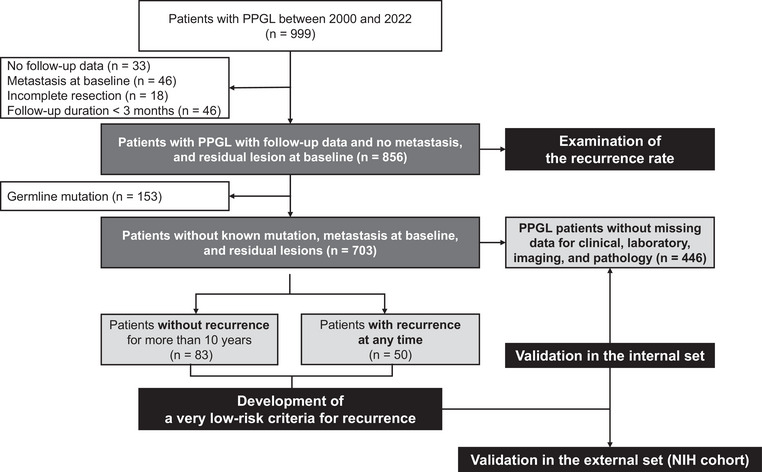
Flow diagram of study subjects. NIH, National Institutes of Health; PPGL, pheochromocytoma and paraganglioma.

From 2000 to 2022, a retrospective cohort of 999 patients diagnosed with PPGL was collected from Seoul National University Hospital (*n* = 483) and Asan Medical Center (*n* = 516), in collaboration with the Korean Adrenal Disorder Study. Research approval was obtained from both the Institutional Review Board of Seoul National University Hospital (No. 2204‐155‐1320) and Asan Medical Center (No. 2022‐1496). Written informed consent was waived due to the retrospective nature of this study. Among them, 33 patients were lost to follow‐up after primary surgery. After excluding patients with metastasis at diagnosis (*n* = 46), incomplete surgical resection (*n* = 18), and a follow‐up duration of less than 3 months (*n* = 46), we analyzed the recurrence rate in 856 patients during the follow‐up duration of 53.0 months (interquartile range [IQR] 22.7–95.8) (Fig. 1).

Next, 153 patients harboring pathogenic germline mutations associated with high‐risk recurrence factors were excluded. The detailed genetic testing results of our cohorts have been reported in a previous publication [16]. In summary, 627 out of 999 patients underwent genetic testing for germline pathogenic variants (PVs). Although the gene panels varied among patients, they consistently included key susceptibility genes such as *SDHx, FH, VHL, RET, NF1, TMEM127, and MAX*. Among these, the *RET* variant was most frequent (8.0%), followed by *VHL* (7.8%), *SDHB* (5.9%), *NF1* and *SDHD* (1.9% each), *SHDC* (1.4%), *SDHA* (1.1%), and *MAX* and *TMEM127* (0.5% each). The frequencies of *FH* and *KIF1B* were 0.3% and 0.2%, respectively.

From the remaining cohort of 703 patients, 50 were diagnosed with recurrent disease during the follow‐up period, whereas 83 patients were followed for over 10 years without any evidence of recurrence. These two subgroups formed the “development set,” designed to establish criteria for very low risk of recurrence. By analyzing baseline characteristics of patients without recurrence for more than 10 years and those who experienced recurrence at any point, we identified features that differed significantly between the two groups. To validate these criteria for very low risk of recurrence, we established a validation cohort of patients with non‐hereditary PPGL who exhibited no metastasis at initial diagnosis and prior to surgery, underwent complete surgical resection, and possessed comprehensive clinical, laboratory, imaging, and pathology data, including pheochromocytoma of the adrenal gland scaled score (PASS) or grading system for adrenal pheochromocytoma and paraganglioma (GAPP) scores (*n* = 446) (Fig. 1). When discrepancies arose between the GAPP score and the PASS, the higher score was chosen to characterize the tumor.

### Validation of the National Institutes of Health (NIH) dataset

Patients who met the very low‐risk criteria for recurrence were evaluated for recurrence using the National Institutes of Health (NIH) *Eunice Kennedy Shriver* National Institute of Child Health and Human Development cohort (protocol 00‐CH‐0093), which included 994 patients with PPGL from 2000 to 2024.

### Data collection

PPGL diagnosis was confirmed through histopathological examination of resected tumors. Biochemical profiling, which included analysis of plasma or urine metanephrines along with imaging studies such as computed tomography (CT) and ^123^I‐metaiodobenzylguanidine (^123^I‐MIBG), facilitated the diagnosis of unresectable tumors.

Clinical data, including age and sex, were extracted from the electronic medical records. Tumor localization and size were determined from thoracic, abdominal, and pelvic CT scans, along with head and neck magnetic resonance imaging. Metastatic or multifocal PPGL was assessed using either ^123^I‐MIBG scan/single photon emission computed tomography or positron emission tomography (PET) with ^68^Ga‐labeled DOTA0‐Tyr3 octreotide (DOTATOC‐PET) or ^18^F‐labeled fluorodeoxyglucose (FDG‐PET). According to the 2022 World Health Organization PPGL classification, PCC was identified as adrenal tumors derived from chromaffin cells, whereas PGLs were classified based on location and tumor characteristics as sympathetic abdominal, sympathetic head and neck, and parasympathetic PGL [17]. The presence of tumor cells in non‐chromaffin sites (e.g., bones, lymph nodes, lungs, and liver) was defined as metastasis. Recurrence was identified as a chromaffin tumor that developed at any time in any location, local recurrence at the surgical site, or newly identified metastases [17]. Biochemical phenotypes were determined by measuring plasma or 24‐h urine levels of normetanephrine and metanephrine. A tumor was classified as an adrenergic/noradrenergic‐negative biochemical phenotype when levels of metanephrine and normetanephrine were within the normal range. A tumor was classified as the adrenergic type if there was an elevation in metanephrine above 5% of the sum of metanephrine and normetanephrine values. All other tumors were defined as having the noradrenergic phenotype [18]. Pathological characteristics were classified based on the PASS and GAPP scores. The PASS consisted of 12 histologic parameters, yielding a total score of 20, with a PASS ≥4 considered aggressive. The GAPP score incorporates the Ki‐67 index within its scoring framework, classifying PPGL into well‐differentiated (0–2), moderately differentiated (3–6), and poorly differentiated (7–10) [19]. We defined the pathologically low‐risk group for recurrence as those with a PASS of <4 or a GAPP score of <3.

Postoperative surveillance was carried out in accordance with current guidelines [13, 14] at both centers. Patients had their first postoperative evaluation at 3 months, which included a CT scan and measurements of plasma and/or 24‐h urine levels of normetanephrine and metanephrine. Subsequently, patients were evaluated annually through routine biochemical surveillance, monitoring plasma and/or urinary levels of metanephrine and normetanephrine. Imaging tests were conducted whenever any elevations in biochemical parameters were detected.

### Statistical analysis

Clinical characteristics based on tumor location and recurrence status were assessed using the chi‐square test or Fisher's exact test for categorical variables and the Mann–Whitney test for continuous variables. Cutoff values for continuous variables to predict recurrence were established using the Youden index in the area under the receiver operating characteristic (ROC) curve (AUROC) analysis from the development set [20]. Cox hazard regression analysis was performed on the validation set to evaluate the recurrence risk based on very low‐risk criteria. Data are presented as hazard ratios (HR) and 95% confidence intervals (CIs). For all tests, a *p*‐value threshold of <0.05 was defined as statistically significant. Statistical analyses were conducted using R version 4.1.2 (R Foundation for Statistical Computing).

## Results

### Baseline characteristics

In the entire cohort of 999 patients, 856 had follow‐up data, did not present with metastasis initially or before surgery, and underwent complete surgical resection (Table S1). Of these, 703 were identified as having sporadic PPGL after excluding 153 patients with germline mutations. Table S2 delineates the clinical characteristics of the 703 patients with sporadic PPGL, comparing them based on tumor location. Approximately 73% (510/703) of the patients had isolated PCC, whereas 22.3% (157/703) and 4.3% (30/703) had sympathetic and head and neck paragangliomas (HNPGLs), respectively. The median age at diagnosis was 51 years, with no significant differences between isolated PCC and other locations. The proportion of women was comparable across groups. The median sizes of PCC and PGL were 4.00 and 4.30 cm, respectively, without significant differences. Nonfunctioning PPGL was observed in 9.9% of patients, being more common in the PGL group than in the PCC group (25.0% vs. 6.2%, *p* < 0.001). PASS data were available for 58.0% (*n* = 411) of patients, with a median score of 3, whereas GAPP scores were available for 32.3% (*n* = 227) of patients, with a median score of 2. The recurrence rate was 7.1%, and the overall mortality rate was 5.4%.

### Recurrence rate in patients with total and sporadic PPGL according to follow‐up period

Among a total of 856 patients with PPGL who had neither metastasis at baseline nor residual lesions after surgery, 94 patients (11.0%) experienced recurrence over a follow‐up period of 53.0 months (range 3.0–363.0, IQR 22.7–95.8) (Figs. S1a and S2a,b). The incidence of new events in these patients was 4.2% (36/856) within 5 years, 3.5% (30/856) between 5 and 10 years, 2.2% (19/856) between 10 and 15 years, and 1.1% (9/856) after 15 years from primary surgery, respectively. Among the 94 patients with recurrence, 38.3% (36/94), 70.2% (66/94), and 90.4% (85/94) experienced recurrence within 5, 10, and 15 years of the primary surgery, respectively. Nine patients (9.6%) developed recurrence more than 15 years after primary surgery.

Among 703 patients without a known mutation, baseline metastasis, or residual lesions, 50 (7.1%) experienced recurrence over a median follow‐up period of 50.7 months (range 3.0–363.0, IQR 22.4–88.5) (Figs. S1b and S2c,d). Additionally, a total of 100 patients (14.2%) were followed for more than 10 years (Fig. S3). Recurrence was identified through routine biochemical follow‐up in 41 patients (82.0%), whereas 9 patients (18.0%) presented with symptoms/signs that prompted re‐evaluation. Of the 50 recurrence events, 21 were locoregional recurrences or metachronous PPGLs, which included contralateral adrenal tumors and PGLs at different sites. Fourteen cases involved distant metastasis, whereas the remainder had both local recurrence and distant metastasis. Among the 50 patients with recurrence, 25 (50.0%) underwent reoperation, 8 (16.0%) received nonoperative treatments such as systemic ^131^I‐MIBG radiotherapy, local radiotherapy, or chemotherapy, and the remaining patients either received no further treatment or had insufficient data. Ultimately, 8 out of the 50 cases resulted in death. Of the 703 patients, 384 (54.6%) were considered adherent to surveillance, defined as (1) patients who completed ≥10 years of follow‐up, (2) those still being monitored at the time of analysis, or (3) those who had been under routine follow‐up until death. In contrast, 222 patients (31.6%) had less than 5 years of follow‐up before becoming lost to follow‐up, and 97 patients (13.8%) had 5–10 years of documented follow‐up but were no longer under surveillance thereafter.

The recurrence rates for sporadic PPGL were 2.6% (18/703) within 5 years, 2.1% (15/703) between 5 and 10 years, 1.7% (12/703) between 10 and 15 years, and 0.7% (5/703) after 15 years. Among the 50 recurrence events, 36.0% (18/50), 66.0% (33/50), and 90.0% (45/50) were detected within 5, 10, and 15 years after surgery, respectively.

To more accurately represent conditional risk based on ongoing follow‐up, we calculated recurrence rates using only patients who remained under surveillance during each time interval as the denominator. In this analysis, the recurrence rates for sporadic PPGL were 4.6% (18/391) within 5 years, 7.1% (15/212) between 5 and 10 years, 15.8% (12/76) between 10 and 15 years, and 20.8% (5/24) after 15 years. Although these conditional rates are higher due to a significant decline in follow‐up numbers, the absolute number of late recurrences remained low. When analyzing patients with PCC and PGL separately, we found that recurrence within 10 years was significantly more common in patients with sporadic PGL compared to those with sporadic PCC (84.7% vs. 59.4%, *p* = 0.047) (Fig. S2d). Furthermore, all patients with sporadic PGL experienced recurrence within 15 years (Fig. S2d).

### Characteristics of very low‐risk group for recurrence

To identify very low‐risk criteria for recurrence, characteristics of patients without recurrence over 10 years (*n* = 83) were compared with those who experienced recurrence at any point (*n* = 50). The median follow‐up duration of 83 patients without recurrence was 142.4 months (range 121.1–325.5, IQR 132.4–175.4, Fig. S4). Patients without recurrence were significantly older at diagnosis (50 vs. 42 years, *p* = 0.001) (Table 1, Tables S3 and S4). The proportion of women and mass locations did not differ between the two groups. The maximum tumor size in PCC was smaller in the non‐recurrence group (4.5 vs. 6.1 cm, *p* = 0.012). Bilateral PCC, multifocal PGLs, and biochemical phenotype showed no significant differences between the groups. Data regarding PASS and GAPP scores were available in 45.8% and 15.7% in non‐recurrence group and 54.0% and 30.0% in recurrence group. The proportion of patients with a PASS <4 was higher in the non‐recurrence group compared to the recurrence group (60.5% vs. 25.9%, *p* = 0.006). Despite no statistical significance, the proportion of patients with a GAPP score <3 was numerically higher in the non‐recurrence group compared to the recurrence group (69.2% vs. 46.7%). Additionally, the proportion of patients with a PASS score <4 or a GAPP score <3 was notably higher in the non‐recurrence group compared to the recurrence group (55.3% vs. 25.0%, *p* = 0.027).

**Table 1:** Baseline characteristics of patients without recurrence over 10 years and those with recurrence at any time.

	Patients without recurrence (*n* = 83)	Patients with recurrence (*n* = 50)	*p*
Age at diagnosis, years	50 (42–57)	42 (29–51)	0.001
Female	48/83 (57.8%)	27/50 (54.0%)	0.700
Location			
PCC	64/83 (77.1%)	37/50 (74.0%)	0.400
sPGL	17/83 (20.5%)	9/50 (18.0%)	
HNPGL	2/83 (2.4%)	3/50 (6.0%)	
PCC, sPGL	0/83 (0.0%)	1/50 (2.0%)	
Size, cm	4.7 (3.0–6.0)	6.0 (3.9–7.8)	0.150
PCC (*n* = 101)	4.5 (3.0–6.0)	6.1 (4.5–8.0)	0.012
PGL (*n* = 26)	5.5 (4.3–6.5)	4.6 (3.4–6.0)	0.666
Bilateral PCC	4/64 (6.3%)	5/38 (13.2%)	0.300
Multifocal PGL	0/19 (0.0%)	1/13 (7.7%)	0.400
Biochemical phenotype			0.600
Adrenergic/Noradrenergic‐negative	5/71 (7.0%)	1/42 (2.4%)	
Adrenergic	38/71 (53.5%)	26/42 (61.9%)	
Noradrenergic	28/71 (39.4%)	15/42 (35.7%)	
PASS data	38/83 (45.8%)	27/50 (54.0%)	
PASS	3.0 (2.0–5.0)	5.0 (3.5–7.0)	0.013
PASS <4	23/38 (60.5%)	7/27 (25.9%)	0.006
GAPP data	13/83 (15.7%)	15/50 (30.0%)	
GAPP score	2.0 (1.0–3.0)	3.0 (0.0–5.0)	0.596
GAPP score <3	9/13 (69.2%)	7/15 (46.7%)	0.500
PASS or GAPP data	38/83 (45.8%)	28/50 (56.0%)	
PASS <4 or GAPP score <3	21/38 (55.3%)	7/28 (25.0%)	0.027
Disease‐free duration, months	142 (132–175)	74 (40–138)	<0.001

*Note*: Data are displayed as *n*(%) or median (IQR). Disease‐free duration = period from the initial surgery to the time of recurrence.

Abbreviations: GAPP, grading system for adrenal pheochromocytoma and paraganglioma; HNPGL, head and neck paraganglioma; IQR, interquartile range; PASS, pheochromocytoma of the adrenal gland scaled score; PCC, pheochromocytoma; PGL, paraganglioma; sPGL, sympathetic paraganglioma.

When analyzing patients with PCC and PGL separately, we observed significant differences in age, tumor size, and the proportion of patients with a PASS <4 or a GAPP score <3 between recurrence and non‐recurrence groups only among those with PCC (Table S3). No such differences were found in patients with PGL (Table S4). After excluding patients with bilateral PCC or multiple PGL (Table S5), we found significant differences in age and PCC size, along with a marginally significant difference in the proportion of patients with a PASS <4 or a GAPP score <3 between the recurrence and non‐recurrence groups. Furthermore, after excluding patients with HNPGLs (Table S6), significant differences in age, PCC size, and the proportion of patients with a PASS <4 or a GAPP score <3 persisted between the recurrence and non‐recurrence groups.

**Fig. 2:**
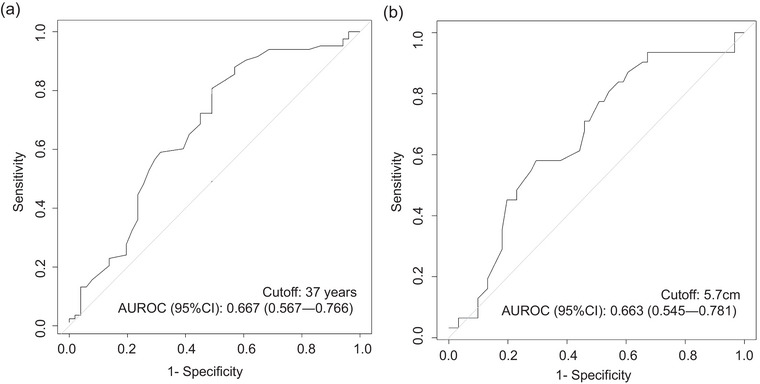
Receiver operating characteristic curve analysis for recurrence based on (a) age and (b) size of pheochromocytoma. AUROC, area under the receiver operating characteristic curve; CI, confidence interval.

In the ROC curve analysis (Fig. 2a), the optimal cutoff age for diagnosis was determined to be 37 years (AUROC: 0.667, 95% CI: 0.567–0.766). This value was rounded to 40 years for simplicity. The ROC curve analysis to determine the optimal size cutoff for recurrence yielded no statistically significant threshold for total PPGL or PGL alone. However, a significant cutoff point emerged when the analysis was restricted to PCC, revealing a size of 5.7 cm (AUROC: 0.663, 95% CI: 0.545–0.781) (Fig. 2b). For consistency with previous studies [12, 21] and to simplify, this size was rounded to 6.0 cm. The prevalence of PASS ≥4 tumors in recurrent PPGL remained higher in patients older than 40 years (76.9% vs. 36.7%, *p* = 0.022) and in tumors ≥6 cm (85.7% vs. 37.5%, *p* = 0.011).

Based on these findings, the very low‐risk criteria for recurrence are an age at diagnosis >40 years, PCC size <6 cm, and pathology criteria including PASS <4 or a GAPP score <3.

### Internal validation

The internal validation set consisted of 446 patients with comprehensive clinical, laboratory, imaging, and pathological data (Fig. 1). The baseline characteristics of the internal validation set (*n* = 446) and patients with missing data (*n* = 257) were detailed in Table S7, Table S8 for patients with PCC, and Table S9 for patients with PGL. Cox regression analysis of the validation set (Table 2) demonstrated that age at diagnosis >40 years (HR: 0.36, 95% CI: 0.17–0.76, *p* = 0.008), a PCC size <6 cm (HR: 0.43, 95% CI: 0.19–0.98, *p* = 0.046), and pathology criteria of PASS <4 or GAPP score <3 (HR: 0.37, 95% CI: 0.16–0.89, *p* = 0.026) significantly correlated with a reduced risk of recurrence.

**Table 2:** Cox proportional hazards regression analysis of recurrence risk in patients from the internal validation cohort (*n* = 446).

	HR (95% CI)	*p*
Age at diagnosis >40 years	0.36 (0.17–0.76)	0.008
PCC size <6 cm^a^	0.43 (0.19–0.98)	0.046
PASS <4 or GAPP score <3^b^	0.37 (0.16–0.89)	0.026

*Note*: Data are depicted as hazard ratios (95% confidence interval).

Abbreviations: 95% CI, 95% confidence interval; GAPP, grading system for adrenal pheochromocytoma and paraganglioma; HR, hazard ratio; PASS, pheochromocytoma of the adrenal gland scaled score; PCC, pheochromocytoma.

^a^ Only in cases of PCC.

^b^Only in cases with an available PASS or GAPP score.

**Fig. 3:**
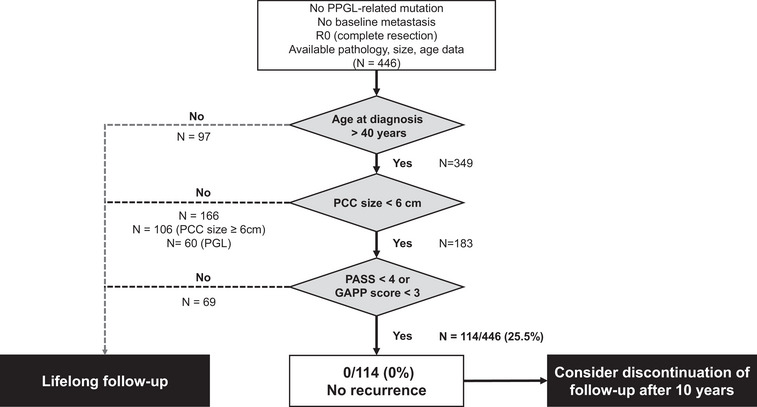
Validation of a very low‐risk group for recurrence in patients with pheochromocytoma and paragangliomas (PPGLs) using the internal validation set. GAPP score, grading system for adrenal pheochromocytoma and paraganglioma score; PASS, pheochromocytoma of the adrenal gland scaled score; PCC, pheochromocytoma; PGL, paraganglioma.

As illustrated in Fig. 3, 97 of 446 (21.7%) patients aged ≤40 were excluded in the first step, and 166 of 349 (47.5%) patients with PCC ≥6 cm or PGL were excluded in the second step. Eventually, 69 of 183 (37.7%) patients who did not meet the pathology criteria (PASS <4 or GAPP score <3) were excluded. Among the remaining 114 of 446 patients (25.5%) who met all very low‐risk criteria for recurrence, including age, location, size, and pathology, none experienced recurrence. The median follow‐up duration in this group was 39.1 (IQR: 16.7–67.8, range: 3.2–175.0) months (Fig. S5).

### External validation from the NIH cohort

Among 994 patients enrolled in the 00CH0093 protocol between 2000 and 2024, 13 patients met all very low‐risk criteria for recurrence, including age, location, and size, but lacked available pathology data (Table S10). These patients, who showed neither evidence of metastasis nor multifocal disease at diagnosis and were identified as sporadic (with negative initial germline genetic testing), had a median age at diagnosis of 50.0 years (range: 44–74 years). None of these 13 patients who met all very low‐risk criteria for recurrence experienced tumor recurrence during the median follow‐up period of 120.0 months (range: 60–204 months).

## Discussion

In this study, the recurrence rate of sporadic PPGLs was 7.1% (50/703), with 36.0% occurring within 5 years, 30.0% between 5 and 10 years, and 24.0% between 10 and 15 years. These findings indicate that recurrences are not confined to the first 10 years after surgery and may provide supportive evidence for considering follow‐up beyond 15 years in appropriately selected patients. Against this background of heterogeneous recurrence risk, we compared 83 patients without recurrence for more than 10 years with those who experienced recurrence at any time (*n* = 50) and identified criteria associated with a very low risk of recurrence: age at diagnosis >40 years, PCC size <6 cm, and a PASS <4 or GAPP score <3. Notably, all of the 114 patients in the internal validation group who met all these very low‐risk criteria for recurrence over a 10‐year follow‐up period did not experience any recurrence. Similarly, 13 patients from the external validation set who met the very low‐risk criteria did not experience any recurrence. Our study is the first to focus on this very low‐risk subgroup for recurrence in patients with sporadic PPGL, suggesting that selected patients may not benefit from prolonged or lifelong follow‐up, based on one of the largest patient cohorts to date.

It is well established that patients with hereditary PPGL require long‐term follow‐up due to a high risk of recurrence [4, 7, 11, 12], whereas the optimal follow‐up strategy for patients with sporadic PPGL remains unclear [4, 8]. Although many recurrences occurred within 5 years [4], late recurrences beyond 10 years have been reported, supporting current recommendations for prolonged surveillance [13, 14]. In our study, 34.0% of recurrence events occurred after 10 years from primary resection in patients with sporadic PPGL, consistent with a previous report [7]. Most recurrences were detected through routine biochemical follow‐up, allowing potentially curative or disease‐modifying interventions. These findings underscore the clinical value of extended follow‐up in selected patients and highlight the importance of identifying those at the opposite end of the risk spectrum—patients who are unlikely to benefit from prolonged surveillance.

Recent guidelines recommend lifelong annual monitoring for high‐risk patients, including younger patients and those with germline variants, large tumors, or PGL [13, 14]. However, no specific subgroups have been identified where follow‐up can be safely discontinued, revealing a gap in the current evidence. Although a single annual test imposes minimal burden, lifelong surveillance can lead to cumulative logistical, psychological, and financial strains. Therefore, current guidelines advocate for a balance between recurrence risk and these considerations [13, 14]. In this study, patients who had no recurrence for more than 10 years were older than 40 years at diagnosis, had a PCC size <6 cm, and had a PASS <4 or a GAPP score <3. Furthermore, there was no recurrence observed in any patient who met all these criteria in either the internal validation set (114/446) or the external validation set (13/994). Given that PPGL is most frequently diagnosed in individuals over 40 years old, it is crucial to alleviate the lifelong medical burden for these selected patients in a very low‐risk category. Accordingly, we propose that patients with sporadic PCC, but not PGL, who are >40 years at diagnosis, have a size <6 cm, and have a PASS <4 or a GAPP score <3 be considered a very low‐risk group and may be candidates for avoidance of routine lifelong follow‐up. Our study is not intended to broadly challenge current guideline recommendations; instead, it aims to provide evidence for identifying a specific subgroup of patients who may be considered for a substantially de‐escalated follow‐up strategy. Therefore, the unique aspect of our study is the identification of very low‐risk criteria for recurrence, which distinguishes it from previous research primarily focusing on high‐risk features of recurrence or metastasis. Besides hereditary predisposition [4, 10, 11, 12], younger age at initial diagnosis [7, 8, 12, 14, 21, 22, 23], extra‐adrenal tumor location [7, 14, 21], larger tumor size [5, 7, 8, 12, 21, 22, 23, 24, 25], highly aggressive pathological features, including PASS and GAPP scores [8, 26, 27], and elevated levels of dopamine and its metabolite, methoxytyramine [12, 23, 28], have been identified as indicators of high recurrence risk. In contrast, our study focuses on defining a very low‐risk group at the opposite end of the risk spectrum, using factors that have previously been associated with high recurrence risk.

The specified age cutoff for high recurrence risk has been reported to range from 35 to 45 years, depending on the studied cohort [12, 21, 29]. Including patients with genetic mutations could significantly influence the age cutoff for predicting recurrence or metastasis. In our cohort of sporadic PPGL cases, an age cutoff of 40 years may be more appropriate for defining a very low‐risk group. The tumor size cutoff for high recurrence risk has been reported as ≥6 cm in some studies [21, 22, 24] but ≥5 cm in others [7, 25]. This variation in tumor size may be due to differences in measurement methods, whether based on imaging or pathology [24]. Formalin fixation of pathological specimens has been reported to induce tumor shrinkage ranging from 45% to 11.4% [30, 31]. Previous studies have demonstrated that the impact of tumor size varies depending on its location, emphasizing the need for distinct size stratification for PCC and PGL [24]. Our study found that PCC <6 cm was associated with a low risk of recurrence, whereas no significant cutoff was identified for PGLs. Consistently, the ROC curves for age and PCC size in our analysis showed only modest discriminatory ability. This highlights that relying solely on single continuous variables is inadequate for guiding surveillance decisions and emphasizes a multifactorial approach, as outlined in our proposed criteria.

Compared to other tumors, the high recurrence risk and genetic background of PPGL necessitate long‐term follow‐up. Therefore, identifying individuals who can be selectively exempted from such extended monitoring is highly meaningful and desired. Our study constitutes the largest multicenter PPGL cohort study in Korea, including nearly 1000 patients. Additionally, our cohort encompasses long‐term follow‐up data spanning over 10 years, providing a sufficient duration to estimate long‐term prognosis. Approximately two‐thirds of the total patients underwent genetic testing, enhancing the data's reliability by excluding patients with PV. To validate our findings, we conducted internal validation using patients with complete clinical data and external validation using NIH cohort data. When we applied our derived criteria to this validation set, we observed a 0% recurrence rate, robustly supporting the reliability of our methodology. However, the follow‐up duration in the validation cohorts might not be long enough to rule out very late recurrences.

Nevertheless, the present study has several limitations. First, despite comprehensive data collection on patients with PPGL, some data about the PASS or GAPP scores were missing. PASS or GAPP scores are not available at every institution due to their complexity and time‐consuming nature. Intra‐ and inter‐observer variability remain inherent limitations of the PASS system, but PASS is still regarded as the best performing pathological method to date [26]. The GAPP scoring system was introduced in 2012 [19], which has likely limited the availability of GAPP data and reduced statistical power. This may explain the nonsignificant difference in GAPP <3, despite a numerical trend. However, due to its increasing use alongside PASS and the high concordance observed in our cohort, GAPP <3 was retained as a supportive, though not essential, low‐risk criterion. Second, in the development cohort, patients with known genetic mutations were excluded, whereas those lacking genetic data were included. Patients with negative genetic test results exhibited slightly higher recurrence rates than those who did not undergo testing, likely reflecting selection bias (8.1% [32/394] vs. 5.8% [18/309]; Fig. S6). However, excluding patients with bilateral PCC or multiple PGL did not affect the reliability of the very‐low‐risk criteria. Additionally, evolving surveillance methodologies may have introduced detection bias, as some late recurrences could represent previously undetected disease. Despite potential referral bias, the high accessibility of these centers, which cover approximately 40% of national PPGL cases, supports the representativeness of our cohort [11]. The predominance of high‐risk patient profiles in both the Korean validation cohort and the NIH cohort from these tertiary academic and government centers may limit the generalizability of the findings, highlighting the need for validation in more diverse healthcare settings. Lastly, given the small number of late recurrences (*n* = 17), recurrent cases at any time were utilized as comparators. Notably, none of the 17 patients with late recurrence met our proposed very low‐risk criteria, which reinforces the validity of this strategy. In addition, the follow‐up duration in the internal validation cohort was relatively limited, which precludes definitive conclusions regarding the absence of very late recurrences beyond 10 years.

In conclusion, our analysis of long‐term outcomes in a large cohort of patients with sporadic PPGL underscores a substantial risk of recurrence, even decades after complete resection. However, our findings indicate that patients with sporadic PCC over the age of 40, with a PCC smaller than 6 cm and a PASS <4 or GAPP score <3, exhibit a very low risk of recurrence. These patients may not benefit from routine lifelong follow‐up. Our study supports a more personalized follow‐up approach based on risk categories that could improve clinical decision‐making, enhance patient quality of life, reduce unnecessary healthcare costs, and alleviate the lifelong surveillance burden in patients with an exceedingly low risk of recurrence.

## Author contributions

Jung Hee Kim and Seung Hun Lee conceptualized the study. Jung Hee Kim, Seung Hun Lee, and Min Jeong Park curated the data and conducted formal statistical analysis. Jung Hee Kim, Seung Hun Lee, Seung Shin Park, Won Woong Kim, Su‐Jin Kim, Yu‐Mi Lee, Kyu Eun Lee, Tae‐Yon Sung, Jae‐Kyung Won, Dong Eun Song, Jung‐Min Koh, Hussam Alkaissi, Sara Talvacchio, Tamara Prodanov, and Karel Pacak conducted the investigations. Jung Hee Kim, Seung Hun Lee, and Min Jeong Park had access to raw data. Jung Hee Kim and Seung Hun Lee designed the methodology. Jung Hee Kim, Seung Hun Lee, and Min Jeong Park were responsible for project administration. Jung Hee Kim, Seung Hun Lee, and Min Jeong Park co‐authored the initial draft of the manuscript. Jung Hee Kim, Seung Hun Lee, Min Jeong Park, Seung Shin Park, Won Woong Kim, Su‐Jin Kim, Yu‐Mi Lee, Kyu Eun Lee, Tae‐Yon Sung, Jae‐Kyung Won, Dong Eun Song, Jung‐Min Koh, Hussam Alkaissi, Sara Talvacchio, Tamara Prodanov, and Karel Pacak revised and edited the manuscript, with data validation from the NIH cohort. All authors approved the final manuscript version. Jung Hee Kim and Seung Hun Lee acquired the funding, but funders did not influence the study design; the collection, management, analysis, or interpretation of data; or the decision to submit the manuscript for publication.

## Conflict of interest statement

The authors declare no conflicts of interest.

## Funding information

This study was funded by a grant from the Korea Health Technology R&D Project through the Korea Health Industry Development Institute (KHIDI), supported by the Ministry of Health and Welfare of the Republic of Korea (Project No. RS‐2024‐00406806, RS‐2025‐02233101), and by a grant from Patient‐Centered Clinical Research Coordinating Center (PACEN) funded by the Ministry of Health & Welfare, Republic of Korea (grant number: RS‐2024‐00397426). This research was also supported in part by the Intramural Research Program of the National Institute of Diabetes and Digestive and Kidney Diseases (NIDDK) and the Eunice Kennedy Shriver National Institute of Child Health and Human Development (NICHD) within the National Institutes of Health (NIH). The contributions of the NIH author(s) are considered works of the United States Government. The findings and conclusions presented in this paper are those of the author(s) and do not necessarily reflect the views of the NIH or the US Department of Health and Human Services. We gratefully acknowledge genetic counselor Hyein Kang for her significant assistance in recruiting patients with PPGLs at SNUH.

## Role of the funding source

The study funders had no influence on the study design, data collection, management, analysis, or interpretation, nor on the decision to submit the manuscript for publication.

## Supporting information




**Table S1**: Baseline characteristics of patients with pheochromocytoma and paraganglioma without metastasis at baseline and residual lesions after surgery (n = 856).
**Table S2**: Baseline characteristics of patients with sporadic PPGL without metastasis at baseline and residual lesions after surgery (n = 703).
**Table S3**: Baseline characteristics of patients with PCC who remained recurrence‐free for over 10 years compared to those who experienced recurrence at any time.
**Table S4**: Baseline characteristics of patients with PGL who remained recurrence‐free for over 10 years compared to those who experienced recurrence at any time.
**Table S5**: Characteristics of patients without recurrence over 10 years and those with recurrence at any time, after excluding those with bilateral PCC or multiple PGLs.
**Table S6**: Characteristics of patients without recurrence over 10 years and those with recurrence at any time, after excluding those with HNPGL.
**Table S7**: Comparison of characteristics between patients in the internal validation set (n = 446) and those excluded (n = 257).
**Table S8**: Comparison of characteristics between patients in the internal validation set and those excluded only among patients with PCC.
**Table S9**: Comparison of characteristics between patients in the internal validation set and those excluded only among patients with PGL.
**Table S10**: Baseline characteristics of the external validation cohort from NIH (n = 13).
**Fig. S1**: Recurrence rate in (a) all patients with PPGL and (b) patients with sporadic PPGL according to the time since primary treatment in patients with PPGL who had no metastasis at baseline and no residual lesions after surgery.
**Fig. S2**: Recurrence rates in all patients with (a) PCC, (b) PGL, (c) sporadic PCC, and (d) sporadic PGL according to the time since primary treatment in patients with PPGLs who had no metastasis at baseline and no residual lesions after surgery.
**Fig. S3**: Distribution of follow‐up period for 703 patients without a known mutation, baseline metastasis, or residual lesion.
**Fig. S4**: Distribution of follow‐up period for 83 patients without recurrence for more than 10 years (N = 83).
**Fig. S5**: Distribution of follow‐up period of internal validation set (N = 114).
**Fig. S6**: Recurrence rates in patients lacking a genetic test and those who underwent genetic test.

## Data Availability

The anonymized individual participant data underlying the reported findings will be available upon reasonable request to the corresponding authors.
