# Supplementary material for: Who can safely discontinue lifelong follow‐up among patients with sporadic pheochromocytoma and paraganglioma?
Source: J Intern Med. 2026 Mar 4;299(5):615–27. doi: 10.1111/joim.70080 (PMC13061095; doi:10.1111/joim.70080)
Supplement: Supplementary file 1 — Table S1: Baseline characteristics of patients with pheochromocytoma and paraganglioma without metastasis at baseline and residual lesions after surgery (n = 856). Table S2: Baseline characteristics of patients with sporadic PPGL without metastasis at baseline and residual lesions after surgery (n = 703). Table S3: Baseline characteristics of patients with PCC who remained recurrence‐free for over 10 years compared to those who experienced recurrence at any time. Table S4: Baseline characteristics of patients with PGL who remained recurrence‐free for over 10 years compared to those who experienced recurrence at any time. Table S5: Characteristics of patients without recurrence over 10 years and those with recurrence at any time, after excluding those with bilateral PCC or multiple PGLs. Table S6: Characteristics of patients without recurrence over 10 years and those with recurrence at any time, after excluding those with HNPGL. Table S7: Comparison of characteristics between patients in the internal validation set (n = 446) and those excluded (n = 257). Table S8: Comparison of characteristics between patients in the internal validation set and those excluded only among patients with PCC. Table S9: Comparison of characteristics between patients in the internal validation set and those excluded only among patients with PGL. Table S10: Baseline characteristics of the external validation cohort from NIH (n = 13). Fig. S1: Recurrence rate in (a) all patients with PPGL and (b) patients with sporadic PPGL according to the time since primary treatment in patients with PPGL who had no metastasis at baseline and no residual lesions after surgery. Fig. S2: Recurrence rates in all patients with (a) PCC, (b) PGL, (c) sporadic PCC, and (d) sporadic PGL according to the time since primary treatment in patients with PPGLs who had no metastasis at baseline and no residual lesions after surgery. Fig. S3: Distribution of follow‐up period for 703 patients without a known mutation, base [file JOIM-299-615-s001.docx]

*Supplementary Table S1:* Baseline characteristics of patients with pheochromocytoma and paraganglioma without metastasis at baseline and residual lesions after surgery (n=856)

|  | Total  (n=856) | Isolated PCC  (n=623) | PGL  (n=233) | p |
| --- | --- | --- | --- | --- |
| Age at diagnosis, years | 50 (38–59) | 49 (38–59) | 53 (38–61) | 0.085 |
| Female | 453/856 (52.9%) | 334/623 (53.6%) | 119/233 (51.1%) | 0.500 |
| Location |  |  |  | <0.001 |
| PCC | 623/856 (72.8%) | 623/623 (100.0%) | 0 (0.0%) |  |
| sPGL | 181/856 (21.1%) | 0 (0.0%) | 185/233 (79.4%) |  |
| HNPGL | 36/856 (4.2%) | 0 (0.0%) | 36/233 (15.5%) |  |
| PCC, sPGL | 14/856 (1.6%) | 0 (0.0%) | 14/233 (6.0%) |  |
| PCC, HNPGL | 1/856 (0.1%) | 0 (0.0%) | 1/233 (0.4%) |  |
| PCC, sPGL, HNPGL | 1/856 (0.1%) | 0 (0.0%) | 1/233 (0.4%) |  |
| Size, cm | 4.2 (2.9–6.0) | 4.0 (2.9–5.9) | 4.5 (2.9–6.0) | 0.700 |
| Bilateral PCC | 67/639 (10.5%) | 55/623 (8.8%) | 12/16 (75.0%) | <0.001 |
| Multifocal PGL | 19/234 (8.1%) | 0.0 (0.0%) | 19/233 (8.2%) | 0.999 |
| Biochemical phenotype |  |  |  | <0.001 |
| Adrenergic/noradrenergic-negative | 72/747 (9.6%) | 36/592 (6.1%) | 36/155 (23.2%) |  |
| Adrenergic | 367/747 (49.1%) | 313/592 (52.9%) | 54/155 (34.8%) |  |
| Noradrenergic | 308/747 (41.2%) | 243/592 (41.0%) | 65/155 (41.9%) |  |
| PASS data | 510/856 (59.6%) | 376/623 (60.4%) | 134/233 (57.5%) |  |
| PASS | 3.0 (2.0–5.0) | 3.0 (2.0–5.0) | 3.0 (2.0–6.0) | 0.300 |
| PASS < 4 | 286/510 (56.1%) | 214/376 (56.9%) | 72/134 (53.7%) | 0.500 |
| GAPP data | 284/856 (33.2%) | 210/623 (33.7%) | 74/233 (31.8%) |  |
| GAPP score | 2.0 (1.0–4.0) | 2.0 (1.0–3.0) | 3.0 (1.0–4.0) | 0.079 |
| GAPP group |  |  |  | 0.140 |
| WD (0-2) | 151/284 (53.2%) | 118/210 (56.2%) | 33/74 (44.6%) |  |
| MD (3-6) | 128/284 (45.1%) | 89/210 (42.4%) | 39/74 (52.7%) |  |
| PD (7-10) | 5/284 (1.8%) | 3/210 (1.4%) | 2/74 (2.7%) |  |
| Recurrence | 94/856 (11.0%) | 68/623 (10.9%) | 26/233 (11.2 %) | 0.999 |
| Disease-free duration, months | 53 (23–96) | 53 (23–99) | 52 (22–92) | 0.400 |
| Death | 43/854 (5.0%) | 28/622 (4.5%) | 15/232 (6.5%) | 0.200 |
| Overall follow-up duration, months | 60 (27–105) | 61 (28–109) | 57 (25–100) | 0.400 |

Data are presented as n (%) or median (IQR)

Disease-free duration=period from the initial surgery to the time of recurrence. GAPP=Grading of Adrenal Pheochromocytoma and Paraganglioma. HNPGL=head and neck paraganglioma. IQR= interquartile range. MD=moderately differentiated. PASS=Pheochromocytoma of the Adrenal Gland Scaled Score. PCC=pheochromocytoma. PGL=paraganglioma. PD=poorly differentiated. sPGL= sympathetic paraganglioma. WD=well-differentiated.

*Supplementary Table S2:* Baseline characteristics of patients with sporadic PPGL without metastasis at baseline and residual lesions after surgery (n=703)

|  | Total patients  (n=703) | Isolated PCC  (n=510) | PGL  (n=193) | p |
| --- | --- | --- | --- | --- |
| Age at diagnosis, years | 51 (41–61) | 51 (41–60) | 54 (41–62) | 0.091 |
| Female | 369/703 (52.5%) | 269/510 (52.7%) | 100/193 (51.8%) | 0.900 |
| Location |  |  |  |  |
| PCC | 510/703 (72.5%) | 510/510(100%) | - | <0.001 |
| sPGL | 157/703 (22.3%) | - | 157/193 (81.4%) |  |
| HNPGL | 30/703 (4.3%) | - | 30/193 (15.5%) |  |
| PCC, sPGL | 6/703 (0.9%) | - | 6/193 (3.1%) |  |
| Size, cm | 4.2 (2.9–6.0) | 4.0 (3.0–6.0) | 4.3 (2.9–6.0) | 0.600 |
| Bilateral PCC | 20/510 (3.9%) | 16/510 (3.1%) | 4/6 (66.7%) | <0.001 |
| Multifocal PGL | 7/193 (3.6%) | - | 7/193 (3.6%) | 0.999 |
| Biochemical phenotype |  |  |  | <0.001 |
| Adrenergic/noradrenergic-negative | 60/606 (9.9%) | 30/486 (6.2%) | 30/120 (25.0%) |  |
| Adrenergic | 305/606 (50.3%) | 257/486 (52.9%) | 48/120 (40.0%) |  |
| Noradrenergic | 241/606 (39.8%) | 199/486 (40.9%) | 42/120 (35.0%) |  |
| PASS data | 411/709 (58.0%) | 300/510 (58.8%) | 116/193 (60.1%) |  |
| PASS | 3.0 (2.0–5.0) | 3.0 (2.0–5.0) | 3.0 (2.0–5.0) | 0.700 |
| PASS <4 | 229/411 (55.7%) | 168/300 (56.0%) | 61/111 (55.0%) | 0.800 |
| GAPP data | 227/703 (32.3%) | 169/510 (33.1%) | 58/193 (30.1%) |  |
| GAPP score | 2.0 (1.0–4.0) | 2.0 (1.0–3.0) | 3.0 (1.0–4.0) | 0.160 |
| GAPP group |  |  |  | 0.200 |
| WD (0–2) | 123/227 (54.2%) | 97/169 (57.4%) | 26/58 (44.8%) |  |
| MD (3–6) | 100/227 (44.1%) | 69/169 (40.8%) | 31/58 (53.4%) |  |
| PD (7–10) | 4/227 (1.7%) | 3/169 (1.8%) | 1/58 (1.7%) |  |
| Recurrence | 50/703 (7.1%) | 37/510 (7.3%) | 13/193 (6.7%) | 0.800 |
| Disease-free duration, months | 51 (22–89) | 52 (22–90) | 49 (23–85) | 0.400 |
| Death | 38/703 (5.4%) | 26/509 (5.1%) | 12/193 (6.2%) | 0.600 |
| Overall follow-up duration, months | 55 (26–98) | 56 (27–103) | 53 (24–92) | 0.300 |

Data are presented as n (%) or median (IQR).

Disease-free duration=period from the initial surgery to the time of recurrence. GAPP=Grading of Adrenal Pheochromocytoma and Paraganglioma. HNPGL=head and neck paraganglioma. IQR=interquartile range. MD=moderately differentiated. PASS=Pheochromocytoma of the Adrenal Gland Scaled Score. PD=poorly differentiated. PGL=paraganglioma. PCC=pheochromocytoma. PPGL=pheochromocytoma and paraganglioma. sPGL=sympathetic paraganglioma. WD=well-differentiated.

***Supplementary table S3.* Baseline characteristics of patients with PCC who remained recurrence-free for over 10 years compared to those who experienced recurrence at any time**

|  | Total PCC  (n = 101) | No recurrence  (n = 64) | Recurrence  (n =37) | p |
| --- | --- | --- | --- | --- |
| Age at diagnosis, years | 47 (38–55) | 50 (42–56) | 40 (28–48) | <0.001 |
| Female | 58/101 (57.4%) | 39/64 (60.9%) | 19/37 (51.4%) | 0.500 |
| Size, cm | 5.0 (3.2–7.0) | 4.5 (3.0–6.0) | 6.1 (4.5–8.0) | 0.012 |
| Bilateral PCC | 9/101 (8.9%) | 4/64 (6.3%) | 5/37 (14.X%) | 0.400 |
| Biochemical phenotype |  |  |  | 0.300 |
| Adrenergic/noradrenergic-negative | 4/90 (4.4%) | 3/57 (5.3%) | 1/33 (3.0%) |  |
| Adrenergic | 51/90 (56.7%) | 29/57 (50.9%) | 22/33 (66.7%) |  |
| Noradrenergic | 35/90 (38.9%) | 25/57 (43.8%) | 10/33 (30.3%) |  |
| PASS data |  |  |  |  |
| PASS | 4.0 (2.0–6.0) | 2.0 (2.0–4.0) | 5.0 (4.0–7.0) | 0.008 |
| PASS <4 | 22/47 (46.8%) | 18/28 (64.3%) | 4/19 (21.0%) | 0.009 |
| GAPP data |  |  |  |  |
| GAPP score | 2.0 (0.74–4.0) | 2.0 (1.0–3.0) | 3.0 (0.0–4.5) | 0.700 |
| GAPP score <3 | 11/20 (55.X%) | 6/9 (66.6%) | 5/11 (45.4%) | 0.500 |
| PASS or GAPP data | 48/101 (47.5%) | 28/64 (43.8%) | 20/37 (54.1%) |  |
| PASS <4 or GAPP score <3 | 21/48 (43.8%) | 17/28 (60.7%) | 4/20 (20.0%) | 0.008 |
| Death | 9/101 (8.9%) | 3/64 (4.7%) | 6/37 (16.X%) | 0.110 |
| Disease free duration, months | 137 (123–171) | 144 (131–179) | 79 (41–153) | <0.001 |

Data are presented as n (%) or median (IQR).

Disease-free duration=period from the initial surgery to the time of recurrence. GAPP=Grading of Adrenal Pheochromocytoma and Paraganglioma. IQR=interquartile range. PASS=Pheochromocytoma of the Adrenal Gland Scaled Score. PCC=pheochromocytoma.

***Supplementary table S4.* Baseline characteristics of patients with PGL who remained recurrence-free for over 10 years compared to those who experienced recurrence at any time**

|  | Total PGL  (n = 32) | No recurrence  (n = 19) | Recurrence  (n =14) | p |
| --- | --- | --- | --- | --- |
| Age at diagnosis, years | 53 (36–60) | 54 (37–60) | 46(30–58) | 0.700 |
| Female | 17/32 (53.1%) | 9/19 (47.4%) | 8/13 (61.5%) | 0.700 |
| Size, cm | 4.9 (3.2–6.3) | 5.5 (4.2–6.5) | 4.5 (1.6–6.0) | 0.200 |
| Multifocal PGL | 1/32 (3.1%) | 0 (0.0%) | 1 (7.7%) | 0.800 |
| Bilateral PCC | 0 (0.0%) | 0 (0.0%) | 0 (0.0%) | - |
| Biochemical phenotype |  |  |  | 0.200 |
| Adrenergic/noradrenergic-negative | 2/23 (8.7%) | 2/14 (14.3%) | 0/9 (0.0%) |  |
| Adrenergic | 13/23 (56.5%) | 9/14 (64.3%) | 4/9 (44.4%) |  |
| Noradrenergic | 8/23 (34.8%) | 3/14 (21.4%) | 5/9 (55.6%) |  |
| PASS data |  |  |  |  |
| PASS | 4.5(2.3–7.0) | 4.0 (2.3–6.5) | 5.0(2.5–8.8) | 0.653 |
| PASS <4 | 8/18 (44.4%) | 5/10 (50.0%) | 3/8 (37.5%) | 0.999 |
| GAPP data |  |  |  |  |
| GAPP score | 1.0 (0.0–4.3) | 1.0 (0.8–1.8) | 2.5 (0.0–5.5) | 0.882 |
| GAPP score <3 | 5/8 (62.5%) | 3/4 (75.0%) | 2/4 (50.0%) | 0.500 |
| PASS or GAPP data | 18/32 (56.3%) | 10/19 (52.6%) | 8/14 (57.1%) |  |
| PASS <4 or GAPP score <3 | 7/18 (38.9%) | 4/10 (40.0%) | 3/8 (37.5%) | 0.999 |
| Death | 3/32 (9.4%) | 1/19 (5.3%) | 2/14 (14.3%) | 0.700 |
| Disease free duration, months | 133 (63–156) | 142 (133–163) | 52 (40–76) | <0.001 |

Data are presented as n (%) or median (IQR).

Disease-free duration=period from the initial surgery to the time of recurrence. GAPP=Grading of Adrenal Pheochromocytoma and Paraganglioma. IQR=interquartile range. PASS=Pheochromocytoma of the Adrenal Gland Scaled Score. PGL=paraganglioma.

*Supplementary Table S5:* Characteristics of patients without recurrence over 10 years and those with recurrence at any time, after excluding those with bilateral PCC or multiple PGLs.

|  | Patients without  recurrence (n = 79) | Patients with  recurrence (n =44) | p |
| --- | --- | --- | --- |
| Age at diagnosis, years | 51 (42–58) | 42 (28–56) | 0.005 |
| Female | 47/79 (59.5%) | 23/44 (52.3%) | 0.600 |
| Location |  |  |  |
| PCC | 60/79 (75.9%) | 32/44 (72.7%) | 0.500 |
| sPGL | 17/79 (21.5%) | 9/44 (20.4%) |  |
| HNPGL | 2/79 (2.6%) | 3/44 (6.9%) |  |
| Size, cm | 4.6 (3.0–6.0) | 5.7 (3.5–7.6) | 0.300 |
| PCC (n=92) | 4.3 (2.9-6.0) | 6.0 (4.2-7.7) | 0.028 |
| PGL (n=26) | 5.5 (4.3-6.5) | 4.6 (3.4-6.0) | 0.666 |
| Biochemical phenotype |  |  | 0.600 |
| Adrenergic/noradrenergic-negative | 5/69 (7.2%) | 1/36 (2.8%) |  |
| Adrenergic | 38/69 (55.0%) | 21/36 (58.3%) |  |
| Noradrenergic | 26/69 (38.7%) | 14/36 (38.9%) |  |
| PASS data | 35/79 (44.3%) | 22/44 (50.0%) |  |
| PASS | 3.0 (2.0–5.0) | 5.0 (3.0–7.0) | 0.023 |
| PASS <4 | 21/35 (60.0%) | 6/22 (27.3%) | 0.033 |
| GAPP data | 12/79 (15.2%) | 12/44 (27.3%) |  |
| GAPP score | 2.0 (1.0–3.0) | 4.0(0.75–5.0) | 0.179 |
| GAPP score <3 | 8/12 (67.0%) | 4/12 (33.3%) | 0.200 |
| PASS or GAPP data | 35/79 (44.3%) | 23/44 (52.3%) |  |
| PASS <4 or GAPP score <3 | 19/35 (54.3%) | 6/23 (26.0%) | 0.056 |
| Disease free duration, months | 142 (131–176) | 69 (37–126) | <0.001 |

Data are presented as n (%) or median (IQR).

Disease free duration=period from the initial surgery to the time of recurrence. GAPP=Grading of Adrenal Pheochromocytoma and Paraganglioma. HNPGL=head and neck paraganglioma. IQR=interquartile range. PASS=Pheochromocytoma of the Adrenal Gland Scaled Score. PCC=pheochromocytoma. PGL=paraganglioma. sPGL=sympathetic paraganglioma

*Supplementary Table S6:* Characteristics of patients without recurrence over 10 years and those with recurrence at any time, after excluding those with HNPGL.

|  | No recurrence  (n = 81) | Recurrence  (n = 47) | p |
| --- | --- | --- | --- |
| Age at diagnosis, years | 50 (42–57) | 39 (28–50) | <0.001 |
| Female | 47/81 (58.0%) | 25/47 (53.2%) | 0.700 |
| Size, cm | 4.7 (3.0–6.0) | 6.0 (4.4–8.0) | 0.034 |
| PCC (n=101) | 4.5 (3.0–6.0) | 6.1 (4.5–8.0) | 0.012 |
| PGL (n=27) | 5.5 (4.3–6.5) | 5.3 (3.7–6.5) | 0.860 |
| Location |  |  | 0.400 |
| PCC | 64/81 (79.0%) | 37/47 (78.7%) |  |
| sPGL | 17/81 (21.0%) | 9/47 (19.1%) |  |
| PCC, sPGL | 0/81 (0.0%) | 1/47 (2.1%) |  |
| Multifocal PGL | 0 (0.0%) | 1/10 (10.0%) | 0.800 |
| Bilateral PCC | 4/64 (6.3%) | 5/38 (13.2%) | 0.400 |
| Biochemical phenotype |  |  | 0.400 |
| Adrenergic/noradrenergic-negative | 5/71 (7.0%) | 1/41 (2.4%) |  |
| Adrenergic | 38/71 (53.5%) | 26/41 (63.4%) |  |
| Noradrenergic | 28/71 (39.4%) | 14/41 (34.1%) |  |
| PASS data |  |  |  |
| PASS | 3.0 (2.0–5.0) | 5.0 (3.0–7.0) | 0.013 |
| PASS <4 | 15/38 (39.4%) | 20/27 (74.1%) | 0.012 |
| GAPP data |  |  |  |
| GAPP score | 2.0 (1.0–3.0) | 3.0 (0.0–5.0) | 0.606 |
| GAPP score <3 | 9/13 (69.2%) | 7/15 (46.7%) | 0.300 |
| PASS or GAPP data | 38/81 (46.9%) | 28/47 (59.6%) |  |
| PASS <4 or GAPP score <3 | 21/38 (55.3%) | 7/28 (25.0%) | 0.012 |
| Death | 4/81 (4.9%) | 8/47 (17.0%) | 0.052 |
| Disease free duration, months | 142 (132–175) | 76 (37–143) | <0.001 |

Data are displayed as n (%) or median (IQR).

Disease free duration=period from the initial surgery to the time of recurrence. GAPP=Grading of Adrenal Pheochromocytoma and Paraganglioma. HNPGL=head and neck paraganglioma. IQR=interquartile range. PASS=Pheochromocytoma of the Adrenal Gland Scaled Score. PCC=pheochromocytoma. PGL=paraganglioma. sPGL=sympathetic paraganglioma

*Supplementary Table* *S7:* Comparison of characteristics between patients in the internal validation set (n=446) and those excluded (n=257)

|  | Internal  Validation group  (n=446) | Patients with missing data  in the low-risk group  (n=257) | p |
| --- | --- | --- | --- |
| Age at diagnosis, years | 53 (43–61) | 50 (37–59) | 0.005 |
| Female | 240/446 (53.8%) | 129/257 (50.2%) | 0.400 |
| Location |  |  | 0.400 |
| PCC | 324/446 (72.6%) | 186/257 (72.4%) |  |
| sPGL | 102/446 (22.9%) | 55/257 (21.4%) |  |
| HNPGL | 15/446 (3.4%) | 15/257 (5.8%) |  |
| PCC, sPGL | 5/446 (1.1%) | 1/257 (0.4%) |  |
| Size, cm | 4.1 (2.9–6.3) | 4.2 (2.9–5.5) | 0.200 |
| Bilateral PCC | 14/329 (4.3%) | 6/187 (3.2%) | 0.600 |
| Multifocal PGL | 6/122 (4.9%) | 1/71 (1.4%) | 0.400 |
| Biochemical Phenotype |  |  | 0.200 |
| Adrenergic/noradrenergic-negative | 33/394 (8.3%) | 27/212 (12.7%) |  |
| Adrenergic | 206/394 (52.3%) | 99/212 (46.7%) |  |
| Noradrenergic | 155/394 (39.3%) | 86/212 (40.6%) |  |
| PASS data | 406/446 (91.0%) | 5/257 (1.9%) |  |
| PASS | 3.0 (2.0–5.0) | 0.0 (0.0–0.0) | 0.022 |
| PASS < 4 | 225/406 (55.4%) | 4/5 (80.0%) | 0.400 |
| GAPP data | 224/446 (50.2%) | 3/257 (1.1%) |  |
| GAPP score | 2.0 (1.0–4.0) | 0.0 (0.0–1.0) | 0.069 |
| GAPP group |  |  | 0.300 |
| WD (0-2) | 120/224 (53.7%) | 3/3 (100%) |  |
| MD (3-6) | 100/224 (44.5%) | 0 (0.0%) |  |
| PD (7-10) | 4/224 (1.7%) | 0 (0.0%) |  |
| Disease-free duration, months | 49 (22–77) | 63 (24–110) | <0.001 |
| Death | 28/445 (6.3%) | 10/257 (3.9%) | 0.200 |
| Overall follow-up duration, months | 53 (24–84) | 66 (27–117) | 0.002 |

Data are presented as n (%) or median (IQR)

Disease-free duration=period from the initial surgery to the time of recurrence. GAPP=Grading of Adrenal Pheochromocytoma and Paraganglioma. HNPGL=head and neck paraganglioma. IQR=interquartile range. MD=moderately differentiated. PASS=Pheochromocytoma of the Adrenal Gland Scaled Score. PCC=pheochromocytoma. PD=poorly differentiated. PGL= paraganglioma. sPGL=sympathetic paraganglioma. WD=well-differentiated.

*Supplementary Table S8:* Comparison of characteristics between patients in the internal validation set and those excluded only among patients with PCC.

|  | PCC of internal validation set  (n=510) | Patient with complete data  (n=324) | Patients with missing data (n=186) | p |
| --- | --- | --- | --- | --- |
| Age at diagnosis, years | 51 (41–60) | 53 (44–61) | 47 (36–56) | <0.001 |
| Female | 269/510(52.7%) | 182/324(56.2%) | 87/186 (47.0%) | 0.051 |
| Size, cm | 4.0 (3.0–6.0) | 4.0 (2.8–6.2) | 4.2 (3.0–5.5) | 0.700 |
| Bilateral PCC | 16/510(3.1%) | 10/324 (3.1%) | 6/186 (3.2%) | 0.999 |
| Biochemical Phenotype |  |  |  | 0.090 |
| Adrenergic/noradrenergic-negative | 30/486 (6.2%) | 14/309 (4.5%) | 16/177 (9.0%) |  |
| Adrenergic | 257/486 (52.9%) | 171/309 (55.3%) | 86/177 (48.6%) |  |
| Noradrenergic | 199/486 (41.0%) | 124/309 (40.1%) | 75/177 (42.4%) |  |
| PASS data |  |  |  |  |
| PASS | 3.0 (2.0–5.0) | 3.0 (2.0–5.0) | 3.0 (0.0–6.0) | 0.800 |
| PASS < 4 | 168/300 (56.0%) | 167/298 (56.0%) | 1/2 (50.0%) | 0.999 |
| GAPP data |  |  |  |  |
| GAPP score | 2.0 (1.0–.0) | 2.0 (1.0–3.0) | 1.0 (0.5–1.5) | 0.239 |
| GAPP group |  |  |  | 0.500 |
| WD (0-2) | 97/169 (57.4%) | 95/167 (56.9%) | 2/2 (100.0%) |  |
| MD (3-6) | 69/169 (40.8%) | 69/167 (41.3%) | 0 (0.0%) |  |
| PD (7-10) | 3/169 (1.8%) | 3/167 (1.8%) | 0 (0.0%) |  |
| Disease-free duration, months | 52 (22–90) | 49 (22–79) | 65 (25–120) | 0.002 |
| Death | 26/509 (5.1%) | 20/323 (6.2%) | 6/186 (3.2%) | 0.200 |
| Overall follow-up duration, months | 56 (27–103) | 53 (26–83.5) | 68 (28–126) | 0.003 |

Data are presented as n (%) or median (IQR)

Disease-free duration=period from the initial surgery to the time of recurrence. GAPP=Grading of Adrenal Pheochromocytoma and Paraganglioma. IQR=interquartile range. MD=moderately differentiated. PASS=Pheochromocytoma of the Adrenal Gland Scaled Score. PCC=pheochromocytoma. PD=poorly differentiated. WD=well-differentiated.

***Supplementary table S9*. Comparison of characteristics between patients in the internal validation set and those excluded only among patients with PGL**

|  | PGL of internal validation set  (n=193) | Patient with complete data  (n=122) | Patients with missing data (n=71) | p |
| --- | --- | --- | --- | --- |
| Age at diagnosis, years | 54 (41–62) | 53 (40–61) | 56 (42–64) | 0.200 |
| Female | 100/193 (51.8%) | 58/122 (47.5%) | 42/71 (59.2%) | 0.200 |
| Size, cm | 4.3 (2.8–6.0) | 4.6 (3.2–6.3) | 3.9 (2.1–5.0) | 0.006 |
| Bilateral PCC | 4/6 (66.6%) | 4/5 (80%) | 0 (0.0%) | 0.700 |
| Multifocal, PGL | 7/193 (3.6%) | 6/122 (4.9%) | 1/71(1.4%) | 0.400 |
| Biochemical Phenotype |  |  |  | 0.600 |
| Adrenergic/noradrenergic-negative | 30/120 (25.0%) | 19/85(22.4%) | 11/35 (31.4%) |  |
| Adrenergic | 48/120 (40.0%) | 35/85(41.2%) | 13/35 (37.2%) |  |
| Noradrenergic | 42/120 (35.0%) | 31/85(36.4%) | 11/35 (31.4%) |  |
| PASS data |  |  |  |  |
| PASS | 3.0 (2.0–5.0) | 3.0 (2.0–5.5) | 0.0 (0.0–0.0) | 0.010 |
| PASS < 4 | 61/111(55.0%) | 58/108 (53.7%) | 3/3 (100.0%) | 0.300 |
| GAPP data |  |  |  |  |
| GAPP score | 3.0 (1.0–4.0) | 3.0 (1.0–4.0) | 0.0 (0.0–0.0) | 0.155 |
| GAPP group |  |  |  |  |
| WD (0-2) | 26/58 (44.8%) | 25/57(43.9%) | 1/1(100.0%) | 0.500 |
| MD (3-6) | 31/58 (53.4%) | 31/57(54.4%) | 0 (0.0%) |  |
| PD (7-10) | 1/58 (1.7%) | 1/54 (1.8%) | 0 (0.0%) |  |
| Disease-free duration, months | 49 (23–85) | 48 (22–71) | 50 (24–103.) | 0.200 |
| Death | 12/193 (6.2%) | 8/122 (6.6%) | 4/71 (5.6%) | 0.999 |

Data are presented as n (%) or median (IQR)

Disease-free duration=period from the initial surgery to the time of recurrence. GAPP=Grading of Adrenal Pheochromocytoma and Paraganglioma. IQR=interquartile range. MD=moderately differentiated. PASS=Pheochromocytoma of the Adrenal Gland Scaled Score. PCC=pheochromocytoma. PD=poorly differentiated. PGL= paraganglioma. WD=well-differentiated.

*Supplementary Table* *S10:* Baseline characteristics of the external validation cohort from NIH (n=13)

|  | **Very low-risk group (n=13)** |
| --- | --- |
| Age at diagnosis, years | 50 (44–74) |
| Sex |  |
| Male | 5/13 (38.5%) |
| Female | 8/13 (61.5%) |
| Location |  |
| PCC | 13/13 (100%) |
| Size, cm | 3.7 (2.5–5.3) |
| Bilateral PCC | 0/13 (0.0%) |
| Biochemical Phenotype |  |
| Adrenergic/noradrenergic-negative | 1/13 (7.7%) |
| Adrenergic | 8/13 (61.5%) |
| Noradrenergic | 4/13 (30.8%) |
| PASS Data | 2/13 (15.4%) |
| PASS < 4 | 2/2 (100.0%) |
| Recurrence | 0 (0.0%) |
| Disease-free duration, months | 120 (60–204) |
| Death | 0 (0.0%) |
| Overall follow-up duration, months | 120 (60–204) |

Data are presented as n (%) or median (IQR).

Disease-free duration=period from the initial surgery to the time of recurrence. IQR=interquartile range. NIH=National Institutes of Health. PASS=Pheochromocytoma of the Adrenal Gland Scaled Score. PCC=pheochromocytoma.

*Supplementary Figure S1.* Recurrence rate in (a) all patients with PPGL and (b) patients with sporadic PPGL according to the time since primary treatment in patients with PPGL who had no metastasis at baseline and no residual lesions after surgery.


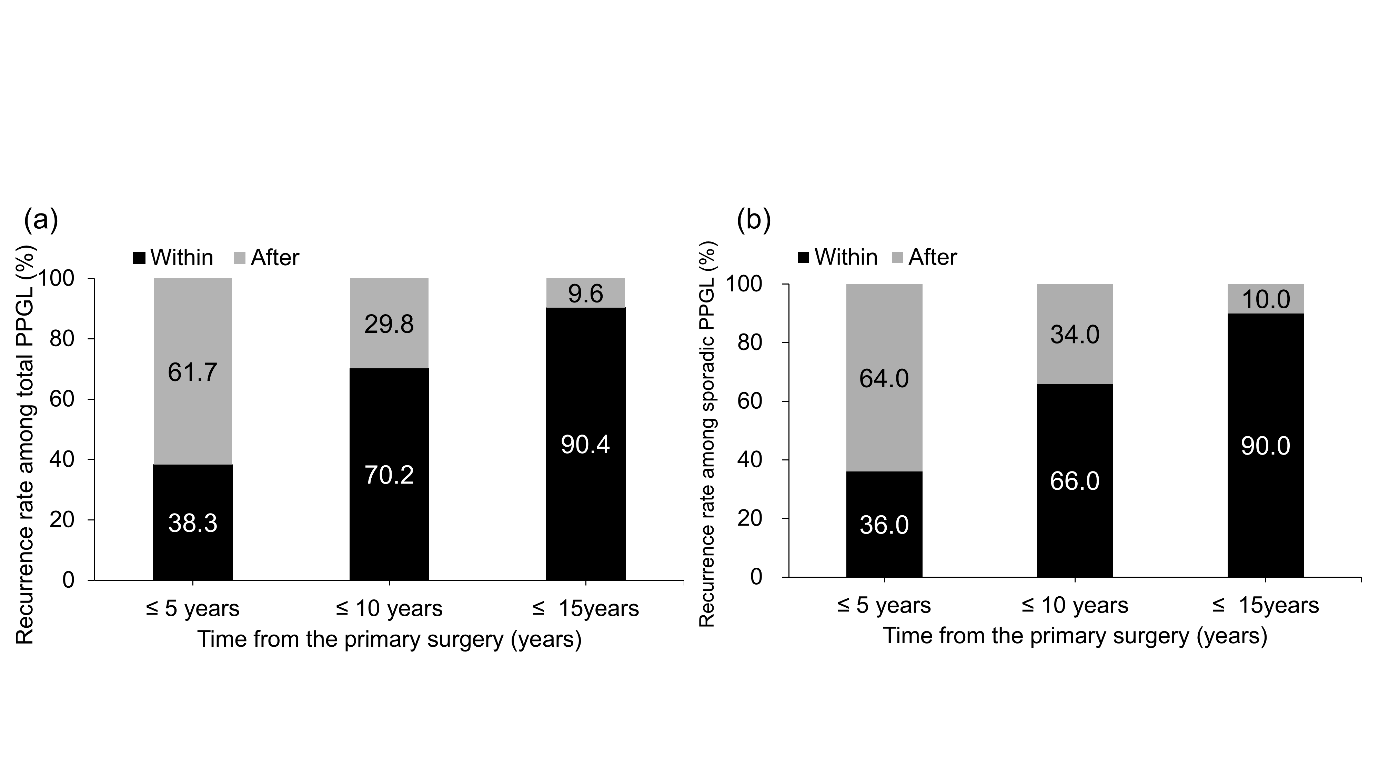


PPGL = pheochromocytoma and paraganglioma.

*Supplementary Figure S2:* Recurrence rates in all patients with (a) PCC, (b) PGL, (c) sporadic PCC, and (d) sporadic PGL according to the time since primary treatment in patients with PPGLs who had no metastasis at baseline and no residual lesions after surgery.


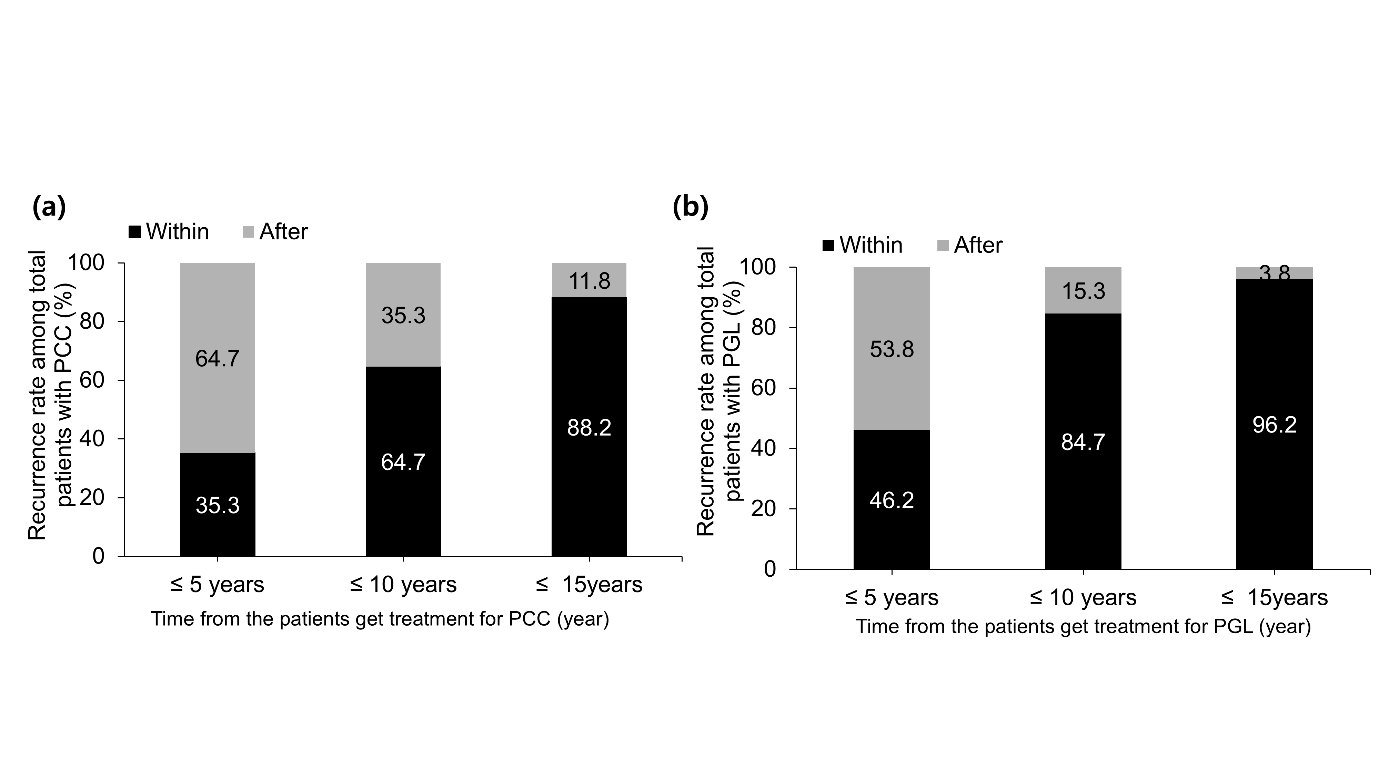

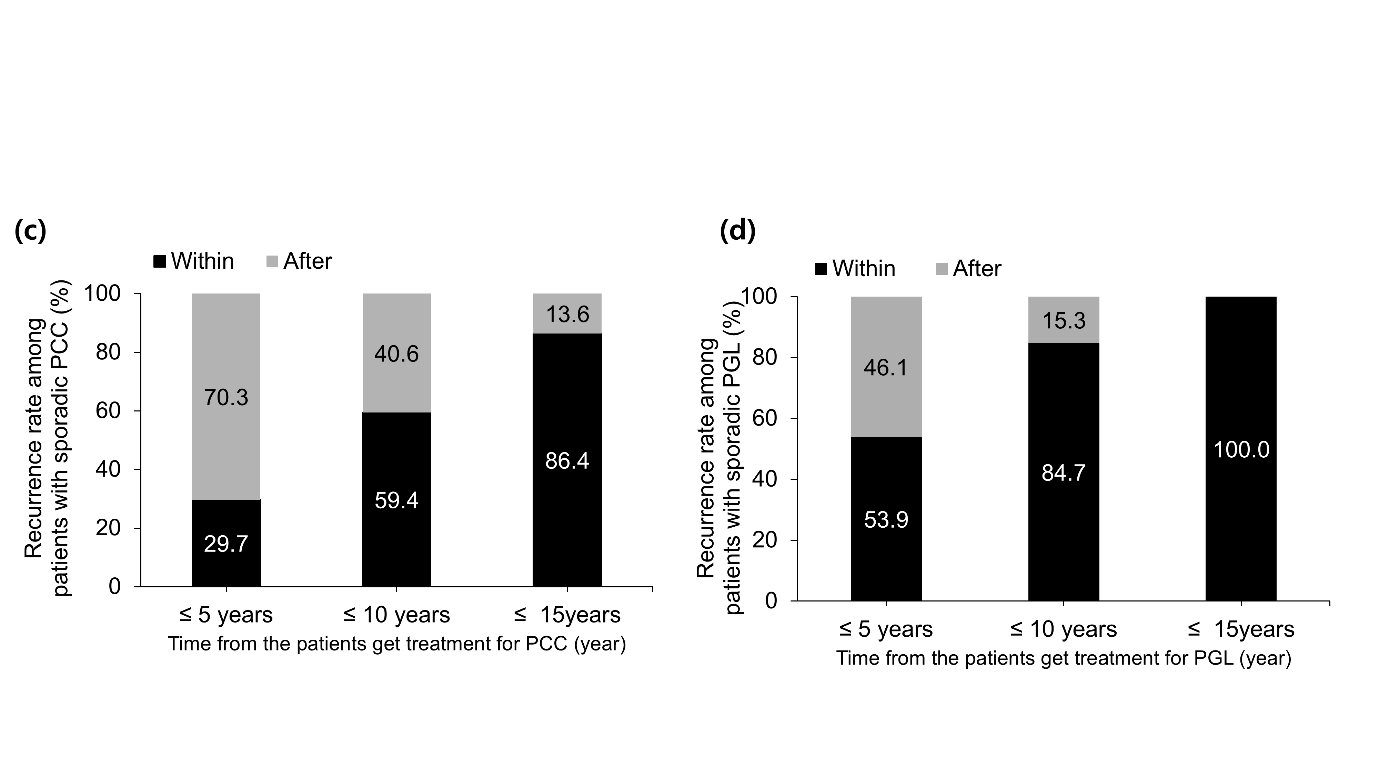


PCC=pheochromocytoma. PGL=paraganglioma. PPGL=pheochromocytoma and paraganglioma.

***Supplementary Figure S3:* Distribution of follow-up period for 703 patients without a known mutation, baseline metastasis, or residual lesion.**

**
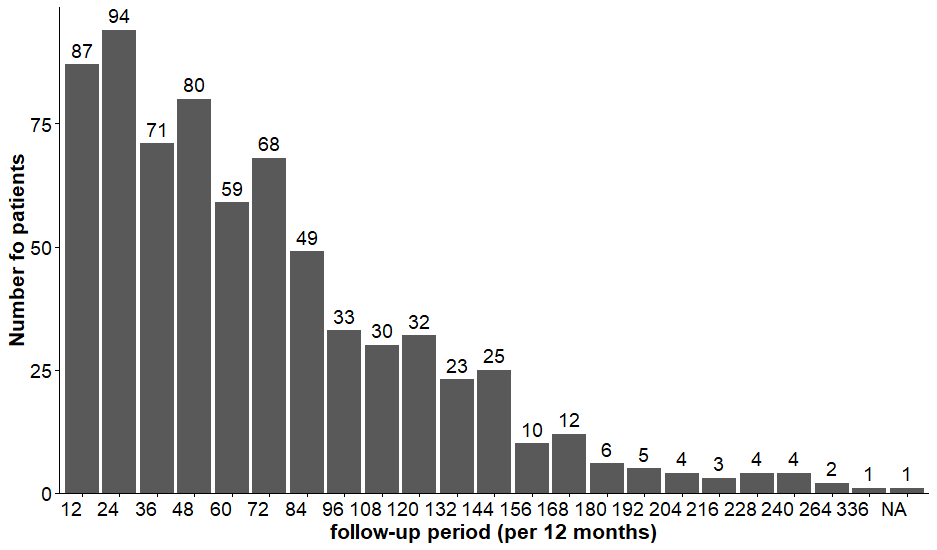
**

***Supplementary Figure S4:* Distribution of follow-up period for 83 patients without recurrence for more than 10 years (N=83)**

**
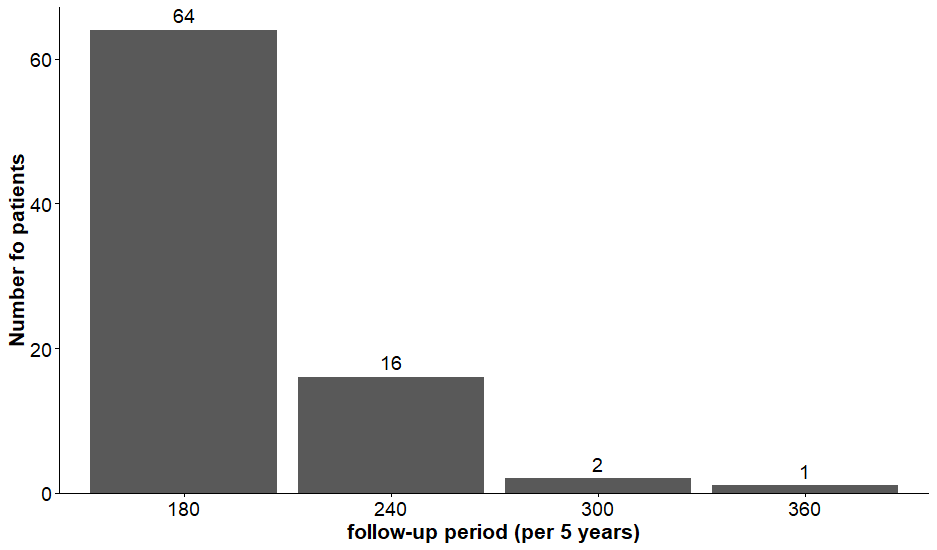
**

***Supplementary Figure S5:* Distribution of follow-up period of internal validation set (N=114)**


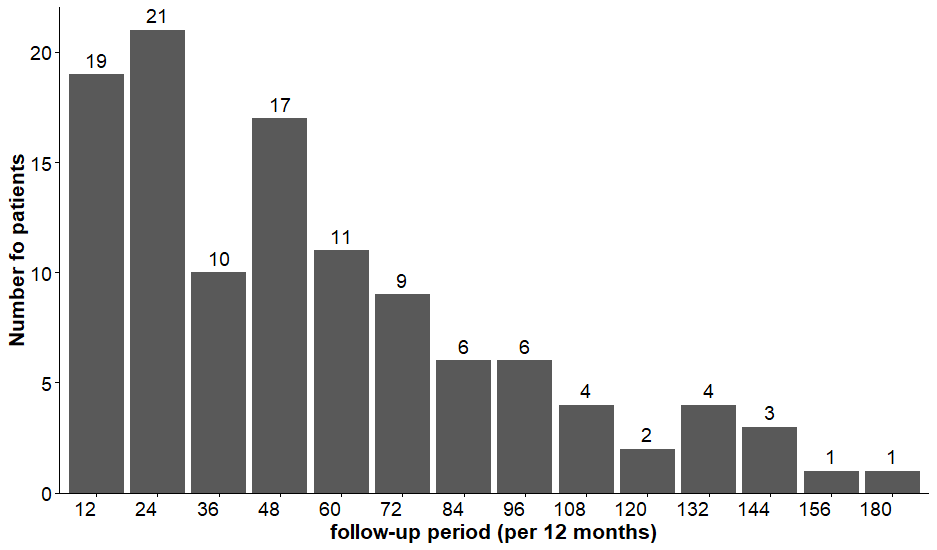


***Supplementary Figure S6.* Recurrence rates in patients lacking a genetic test and those who underwent genetic test.**


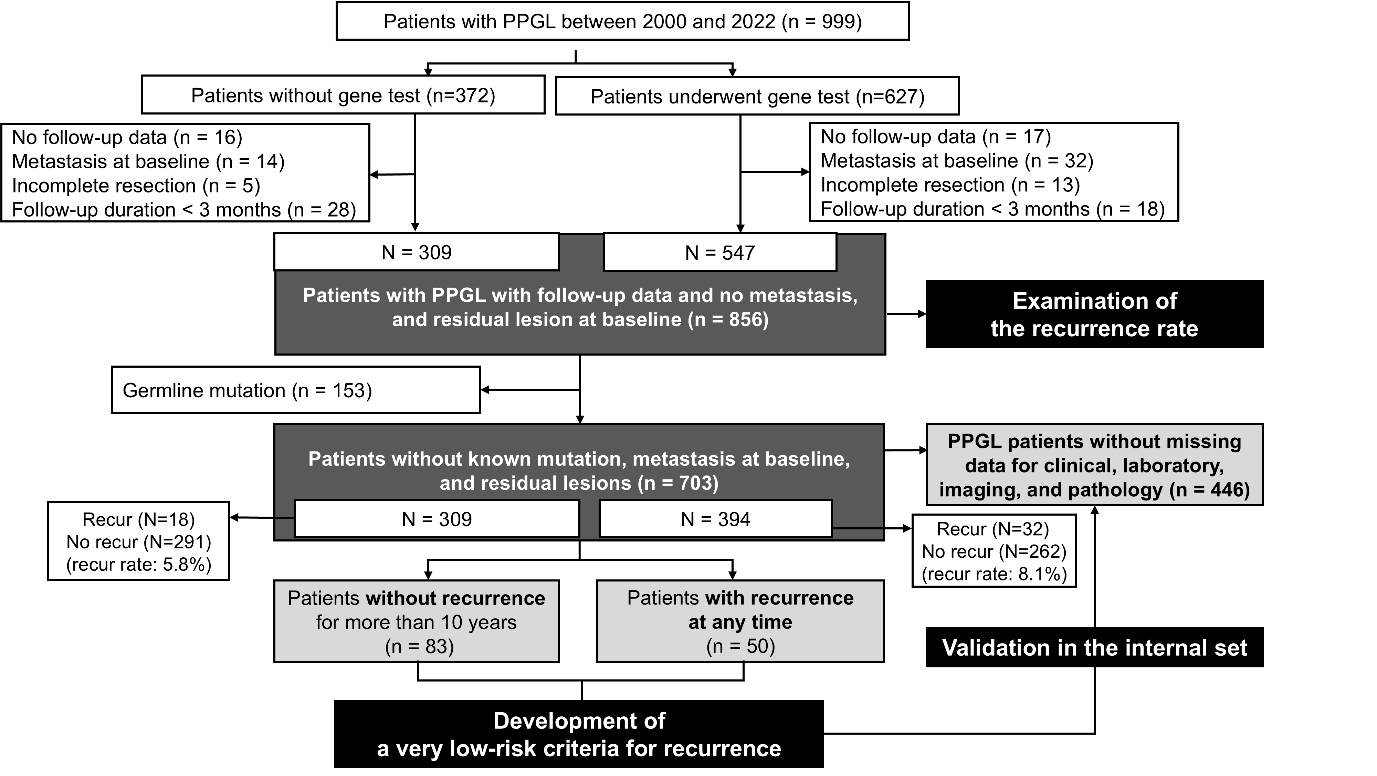


PPGL=pheochromocytoma and paraganglioma.
